# Retraction Note: Carbon dot nanozymes as free radicals scavengers for the management of hepatic ischemia-reperfusion injury by regulating the liver inflammatory network and inhibiting apoptosis

**DOI:** 10.1186/s12951-025-03819-8

**Published:** 2025-11-12

**Authors:** Haoge Geng, Jiayu Chen, Kangsheng Tu, Hang Tuo, Qingsong Wu, Jinhui Guo, Qingwei Zhu, Zhe Zhang, Yujie Zhang, Dongsheng Huang, Mingzhen Zhang, Qiuran Xu

**Affiliations:** 1 Laboratory of Tumor Molecular Diagnosis and Individualized Medicine of Zhejiang Province, Affiliated People’s Hospital, Zhejiang Provincial People’s Hospital, Hangzhou Medical College, Hangzhou, 310014 Zhejiang China; 2 Department of Hepatobiliary Surgery, The First Affiliated Hospital of Xi’an Jiaotong University, Xi’an, 710061 Shaanxi China; 3 The Second Clinical Medical College, Zhejiang Chinese Medical University, Hangzhou, 310053 Zhejiang China; 4 Qingdao Medical College, Qingdao University, Qingdao, 266071 Shandong China; 5 School of Basic Medical Sciences, Xi’an Jiaotong University, Xi’an, 710061 Shaanxi China

**Retraction Note: Journal of Nanobiotechnology (2023) 21:500**

10.1186/s12951-023-02234-1

The authors have retracted this article. After publication, the authors found some errors in the image selection for Figs. 4 A, S3A and S5A, which resulted in duplicated subpanels. Additionally, the authors used L-02 cells as a model of hepatocytes; however, this cell line has been reported to be contaminated with HeLa cells and therefore does not represent normal liver cells.

The authors have repeated the experiments using a different liver cell line and intend to resubmit a revised manuscript for peer review.

All authors agree to this retraction.

